# Editorial: Optical technologies for disease diagnosis and therapy in deep tissues

**DOI:** 10.3389/fbioe.2022.1012949

**Published:** 2022-09-09

**Authors:** Rui-Lin Liu

**Affiliations:** School of Pharmacy, Xuzhou Medical University, Xuzhou, China

**Keywords:** optical imaging, optoacoustic imaging, interactions, therapeutic strategies, nano drug

Optical technology has aroused great interest in biological and medical fields (Yang et al., 2020). The challenge now is how to improve the tissue penetration, signal-to-noise ratio, and spatial resolution in deep tissues, as well as broaden their potential applications (Liu et al., 2020; Zhang et al., 2022). This Research Topic is aimed to summarize the recent optical imaging technologies for diagnosis and treatment of various diseases, which will supply complementary views from nanomedicine researchers into arising ideas in biomedical imaging and nanoprobe-related disease treatment.

It is pleased to see that this Research Topic collects seven articles, which report both the latest research advances of optical technologies for disease diagnosis and therapy, as well as intelligent nano drugs. In content, these articles can be divided into three categories.

The first category is the Fluorescence imaging (FI) and optoacoustic imaging (OA) in animal models. The FI technique with high sensitivity and noninvasiveness can be used for visualizing the vascular system, lymphatic system, various tumor tissues, real-time blood flow and others. Here, Liu et al. review recent advances in near-infrared (NIR) fluorescence imaging for the detection and assessment of pulmonary fibrosis. In this review, they systematically summarize various pulmonary fibrosis-related imaging techniques involving collagen, oxidative stress, inflammation, and typical biomarkers. The OA has a high sensitivity and excellent spatial resolution and its imaging depth can up to centimeters. In particular, the optoacoustic-based multimodal imaging can obtain multiplex molecular, structural, and functional imaging information in deep tissue. Shi et al. summarize various contrast agents such as chemical dyes and nanoparticles for multimodal optoacoustic brain imaging in small animals, including OA/FI, OA/computed tomography, OA/magnetic resonance imaging (MRI), OA/positron emission tomography, and OA/MRI/Raman imaging.

The second category is the interactions between optical probes and living systems. A thorough understanding of the interaction between optical nanoprobes and living systems is of great significance for the development and clinical transformation of multifunctional contrast agents. Typically, Wang et al. present the effects of InP/ZnS-COOH quantum dots (QDs) and InP/ZnS-NH_2_ QDs on the 1,2-Dipalmitoyl-sn-glycero-3-phosphocholine/1,2-dipalmitoyl-sn-glycero-3-phosphoglycerol sodium mixed monolayer. This work offer helpful results for thorough comprehension of the influences of the amino or carboxyl group modified InP/ZnS QDs on the surface of lung surfactant membrane. This is mainly due to the widespread applications of the InP/ZnS QDs in biomedical fields.

The third category is optical related therapeutic strategies and fabrication of intelligent nano drugs. The techniques of this category are mainly uses optical related techniques and advanced nanocarries to treat various diseases. Zhu et al. use the zebrafish model to investigate the influences of Zn^2+^ from Ti-NW-Zn surfaces on angiogenesis and osteogenesis, the results of the HUVECs and MC3T3-E1s *in vitro* revealed that the relationship between angiogenesis and osteogenesis. Song et al. synthesize Ce/Gd@hydroxyapatite (HA) by co-doping Ce^3+^ and Gd^3+^, and then incorporate Ce/Gd@HA nanoparticles into polylactic-co-glycolic acid (PLGA) for promoting osteoblast viability, repairing tibia of the rats and enhancing MRI. Xi et al. review the classification of genetic engineering (i.e., gene therapies) for cancer treatment, and reveal the effect of the nano-drug carrier in ameliorating the efficiency of gene editing. Liao and Niu explore latest applications of the CD47-SIRPα for anticancer therapy, and discuss the combination between CD47-SIRPα and other targeting drugs, as well as CD47-SIRPα and other treatment methods. Note that the systemic injection of CD47-SIRPα can produce serious adverse reactions during tumor immunotherapy owing to its nonspecific targeting property. However, this bottleneck will provides an enormous opportunity for the development of nanomedicine.

In short, this Research Topic should be of interest to readers in the fields of optics, nanomedicine, smart nano drugs, and biomaterial subjects. It is hope that this special Research Topic will promote the scientific progress of optical imaging and optical related diagnosis and treatment technology, as well as their practical applications in biology and nanomedicine.
